# Simple, Reproducible, and Efficient Clinical Grading System for Murine Models of Acute Graft-versus-Host Disease

**DOI:** 10.3389/fimmu.2018.00010

**Published:** 2018-01-22

**Authors:** Sina Naserian, Mathieu Leclerc, Allan Thiolat, Caroline Pilon, Cindy Le Bret, Yazid Belkacemi, Sébastien Maury, Frédéric Charlotte, José L. Cohen

**Affiliations:** ^1^Université Paris-Est, UMR_S955, Université Paris-Est Créteil Val de Marne, Créteil, France; ^2^INSERM, U955, Equipe 21, Créteil, France; ^3^APHP, Service d’hématologie Clinique, Hôpital Henri Mondor, Créteil, France; ^4^UPEC, APHP, INSERM, CIC Biothérapie, Hôpital Henri Mondor, Créteil, France; ^5^Université Paris-Est Créteil Val de Marne, APHP, Service d’Oncologie-Radiothérapie, Hôpital Henri Mondor, Créteil, France; ^6^APHP, Hôpital Pitié Salpêtrière, Service d’Anatomopathologie, Paris, France

**Keywords:** acute graft-versus-host disease, mice, clinical grading, classification, murine models, prognostic factor

## Abstract

Acute graft-versus-host disease (aGVHD) represents a challenging complication after allogeneic hematopoietic stem cell transplantation. Despite the intensive preclinical research in the field of prevention and treatment of aGVHD, and the presence of a well-established clinical grading system to evaluate human aGVHD, such a valid tool is still lacking for the evaluation of murine aGVHD. Indeed, several scoring systems have been reported, but none of them has been properly evaluated and they all share some limitations: they incompletely reflect the disease, rely on severity stages that are distinguished by subjective assessment of clinical criteria and are not easy to discriminate, which could render evaluation more time consuming, and their reproducibility among different experimenters is uncertain. Consequently, clinical murine aGVHD description is often based merely on animal weight loss and mortality. Here, we propose a simple scoring system of aGVHD relying on the binary (yes or no) evaluation of five important visual parameters that reflect the complexity of the disease without the need to sacrifice the mice. We show that this scoring system is consistent with the gold standard histological staging of aGVHD across several donor/recipient mice combinations. This system is also a strong predictor of survival of recipient mice when used early after transplant and is highly reproducible between experimenters.

## Introduction

Allogeneic hematopoietic stem cell transplantation (allo-HSCT) is the treatment of choice for many malignant hematological disorders, such as acute leukemias or the myelodysplastic syndrome (1). Although this cell-based therapy allows for a good prevention of disease relapse through the eradication of residual leukemic cells by donor T cells, the so-called graft-versus-leukemia effect (GVL) (2), the harmful counterpart of GVL, known as graft-versus-host disease (GVHD), is responsible for the destruction of the recipient’s normal cells and tissues and is a major cause of posttransplant morbidity and mortality. GVHD accounts for 15–30% of deaths after allo-HSCT, and its incidence can be as high as 60–80% of transplant recipients in case of one-antigen HLA-mismatched unrelated donor (3). Acute graft-versus-host disease GVHD (aGVHD) usually occurs during the first 3 months after transplant and typically affects three target organs: skin, intestinal tract, and liver.

To improve the outcome of patients suffering from aGVHD, or even prevent its occurrence, many preclinical studies have focused on this disease over the past decades. Most of preclinical data have emerged from mice models, which are useful to design preventive strategies (4), although they imperfectly reflect the disease observed in humans (5). However, other experimental models such as rat models are also used (6). The indispensible prerequisite to properly assess the efficacy of a therapeutic approach of aGVHD is to be able to efficiently and easily evaluate and grade the intensity and/or severity of this condition throughout the experiment. For the assessment of human aGVHD, a simple and efficient clinical grading system has been widely used for decades (7). Regarding murine aGVHD, the situation is less clear. A rapid analysis of the literature reveals at least two approaches: aGVHD is either described merely through animal weight curve evolution and mortality or evaluated according to a multiplicity of clinical scoring systems that have usually been adapted/modified in each laboratory from the one originally described by Cooke et al. in 1996 (8). To the best of our knowledge, none of these many grading systems that have been described (8–15) (Table 1) has been properly evaluated in terms of reproducibility, consistency with histological findings, prediction of survival, and validity in multiple donor/recipient mice genetic combinations. Moreover, most of them share several drawbacks. First of all, even though symptoms related to gut aGVHD, mainly diarrhea, are a major clinical feature of this disease in humans and mice, reflecting a high degree of severity, most of the murine clinical grading systems, including the one reported by Cooke et al., do not include a specific evaluation of these symptoms (8). The main reason for this absence is the fact that these scores were usually developed to capture differences in severity for each clinical parameter and one can easily figure out that quantitating daily stool volume or frequency in mice would be virtually impossible. However, stating for each mouse if diarrhea is present or not is quite easy, by simple examination of the anal area or emission of liquid stool at manipulation. Another reason mentioned to explain the lack of diarrhea evaluation is the fact that some of the other clinical parameters evaluated in the scores, such as weight loss, hunching, or activity, could probably reflect cachexia and is associated with gut aGVHD. However, these clinical signs are not specific at all and seem to be more related to a poor clinical condition, whatever the cause of this weakness. The second limitation of clinical grading systems of murine aGVHD could be the separation of each item into several severity stages, usually scored from 0 to 2. Although, this was originally intended to increase the ability to easily distinguish different stages for each item, just like in the human grading system, it seems that the distinction between the three severity stages is often hard to make and could impact on the reproducibility of the evaluation between two different observers. This is probably due to the fact that, unlike human grading system, most of the items in murine scoring systems are not separated into different stages according to objective measurements but rather thanks to a subjective assessment of their intensity. Finally, the use of a more simple, i.e., binary evaluation of each item (“yes or no”), apart from being less subjective, could also be less time consuming as compared with a stratified classification.

**Table 1:** Main references for acute graft-versus-host disease (GVHD) grading.

Reference	Criteria	Points for each criteria	Total points	Correlation with histological grading
Cooke et al. (8)	Weight loss, posture, activity, fur texture, and skin integrity	0–2	10	No

Anderson et al. (9)	Skin ulcers with different size of alopecia and the site of skin lesion	Skin ulcers less than 1 cm^2^ = 1, between 1 and 2 cm^2^ = 2, and more than 2 cm^2^ = 3 + 0.3 points for each site of skin disease (ears, tails, and paws)	3.9	No

Mutis et al. (XenoGVHD) (14)	Weight loss, mobility, and general appearance	0–2 for mobility; 0, normal fur; 1, ruffled fur; 2, ruffled fur + red swollen skin; and 3, ruffled fur + red swollen skin + patchy alopecia	5 + weight loss was >10%	No

Wilson et al. (15)	Weight loss, posture, activity, fur texture, skin integrity, and diarrhea	0–2	12	No

Castor et al. (11)	Weight loss, posture, activity, fur texture, skin integrity, diarrhea, and occult blood in feces	0–2	14	No

Lai et al. (13)	Weight loss, posture, activity, fur texture, skin integrity, and diarrhea	0–2; for diarrhea no (0) or yes (1)	11	No

Budde et al. (10)	Posture, activity, fur/skin, and diarrhea	0–2	8	No

Doisne et al. (12)	Weight loss, posture, activity, and fur texture	0–2	8	No

Proposed scoring system	Weight loss, hunched posture, fur texture, skin integrity, and diarrhea	No = 0; yes = l	5	Yes

*Parameters taken into account for each type of clinical grading*.

Therefore, we developed and tested against the gold standard of histological evidence of GVHD an adaptation of the murine clinical grading system described by Cooke et al. (8), based on the binary evaluation (absence = 0 or presence = 1) of five essential signs and symptoms of the disease (Table 1) over time: weight loss >10% of initial weight, hunching posture, skin lesions, dull fur, and diarrhea. As compared with the Cooke grading system, we also chose to remove the activity criteria because we found it not only unspecific but also difficult to objectively evaluate in a binary evaluation. This tool allows us to overcome all the abovementioned limitations of the currently used grading systems. Moreover, we show that it is highly reproducible, consistent with histological features of the disease and applicable through a wide variety of genetic combinations. It is also a powerful predictor of mice survival.

## Materials and Methods

### Mice

Wild-type BALB/c (H-2^d^), DBA2 (D2, H-2^d^), C57BL/6 (B6, H-2^b^), C3H (H-2^k^), B6C3HF1 (B6xC3H, H-2^kb^), and B6D2F1 (B6xD2, H-2^bd^) mice were purchased from Harlan Laboratories (Gannat, France) and Charles River Laboratories (Saint-Germain-Nuelles, France). Mice were housed under specific pathogen-free conditions. All experimental protocols were approved by the local ethics committee (authorization no. 11/12/12-5B) and are in compliance with European Union guidelines.

### GVHD and Transplantation Models

Eight- to-twelve-week-old recipient B6C3F1, B6D2F1, C3H, or D2 female mice received a 10-Gy irradiation followed by retro-orbital infusion of 10 × 10^6^ bone marrow (BM) cells (B6 or BALB/c) + 2 × 10^6^ CD3 + (B6 or BALB/c) conventional T cells (Tconv).

T cell suspensions were prepared after mechanical dilacerations of spleens and ammonium–chloride–potassium (ACK) lysis of red blood cells. Around 30% of total splenocytes were CD3^+^ cells. BM cell suspensions were prepared using leg bones as previously described (16–18). Briefly, BM cells were extracted from leg bones by flushing with PBS buffer, filtered, and ACK lysis of red blood cells was performed. For experiments of aGVHD prevention, donor regulatory T cells (Tregs) were obtained as previously described (18) and infused at time of transplant by retro-orbital infusion in a 1:1 Treg/Tconv ratio. To inhibit donor Tregs effect, recipient mice received three intraperitoneal infusions of 500 µg of an anti-TNFR2 monoclonal antibody, as previously described (19). For reproducibility tests, recipient B6C3F1 or B6D2F1 mice received T cells from either B6 donor mice or from previously protected B6C3F1 mice that had undergone primary semi-allogeneic transplantation from B6 mice in the presence of Tregs.

### aGVHD Grading

Acute GVHD clinical score was calculated three times a week. Each of the five following parameters was scored 0 (if absent) or 1 (if present): weight loss >10% of initial weight, hunching posture, skin lesions, dull fur, and diarrhea attested by liquid stool production at time of mice manipulation or its presence at the anal area. Dead mice received a total score of 5 until the end of experiment. Mice were sacrificed in case of weight loss >30% of initial weight or upon reaching the maximal clinical grade (i.e., 5/5). Reproducibility of the grading system was tested by involving three different experimenters on four independent experiments in two different genetic combinations.

### Histology

After mice death or sacrifice, small and large bowel, liver, and skin samples were fixed in 4% formaldehyde solution and embedded in paraffin. For each organ, 5-µm sections were stained with H&E for histological examination. One pathologist analyzed the slides in a blinded fashion to assess the intensity of GVHD. Six parameters were scored for small and large bowel according to a 0- to 5-point scale adapted from Cooke et al. (20) (surface colonocyte lesions or villous blunting, crypt regeneration, crypt epithelial cell apoptosis, crypt loss, lamina propria inflammation, and mucosal ulceration); seven parameters for the liver according a 0- to 3-point scale (portal inflammation, bile ducts lesions, periportal necrosis, endothelialitis, lobular necro-inflammatory activity, zonal necrosis, and sinusoidal lymphocytosis) (20); three parameters for the skin according to a 0- to 3-point scale described by Ferrara et al. (basal cell layer vacuolization, epidermal and follicular dyskeratosis, and epidermal and dermal lymphocytic infiltrate) (21). Scores of each item were added up to provide a total score for each organ, and scores of each target organ were added up to determine a global histological score for each mouse.

### Statistical Analysis

Prism (GraphPad Software) was used for statistical analysis. Kaplan–Meier survival curves were compared using the log-rank test. For analysis of aGVHD clinical grading curves, the area under curve was calculated for each mouse, and then *t*-test or one-way ANOVA with *post hoc* analysis was performed depending on the number of compared variables. Correlation analyses were performed using Spearman’s non parametric correlation test after checking for non-Gaussian distribution of values using the Shapiro–Wilk normality test.

## Results

### The Clinical Grading System Is Consistent with the Histological Evaluation of aGVHD

**Figure 1:**
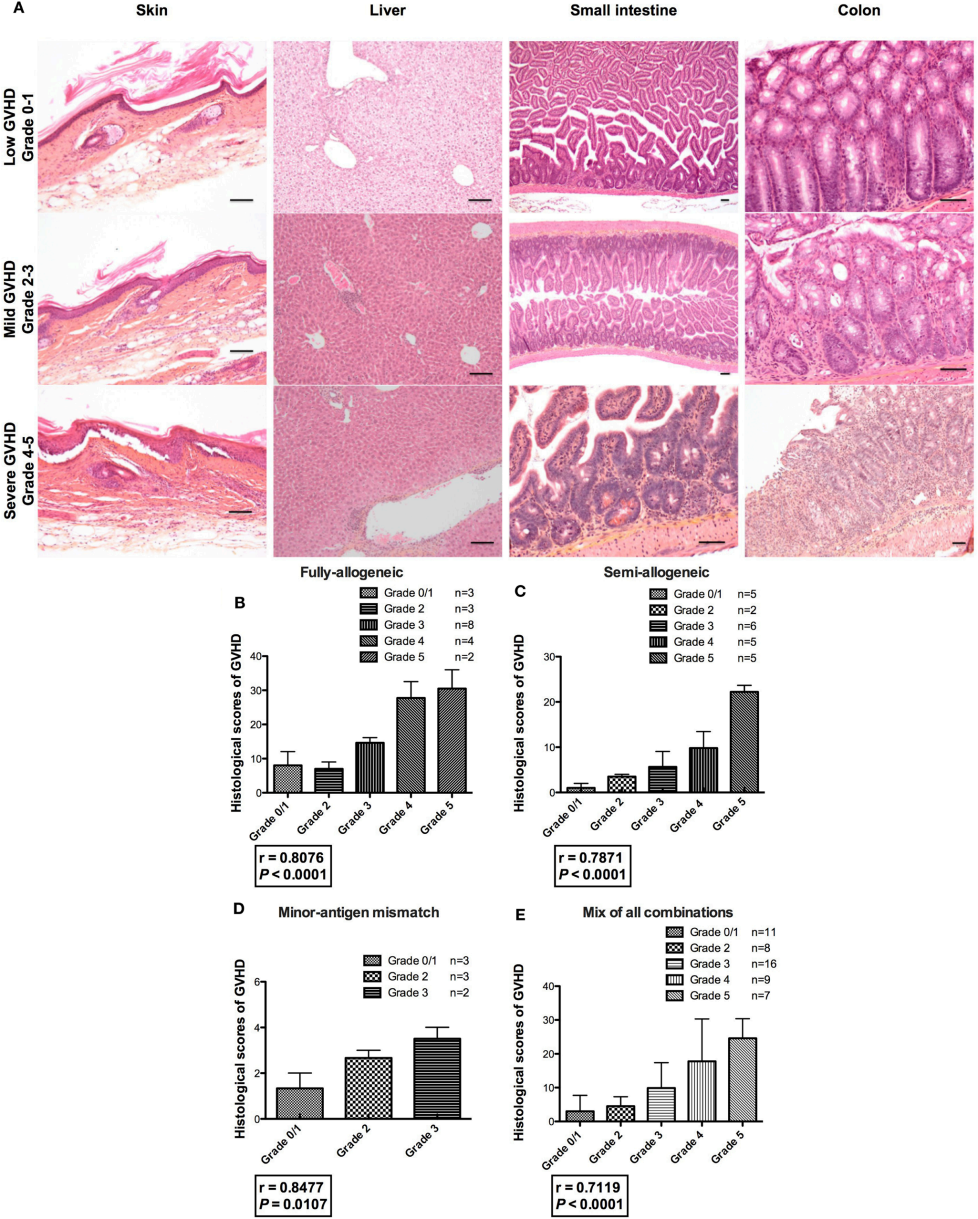
Correlation between clinical and histological scores of acute graft-versus-host disease GVHD (aGVHD). **(A)** Histopathological examination of aGVHD was assessed by a pathologist blinded to the nature of the mice being examined in the skin (100×), liver (100×), small intestine (25×, 25×, and 100×), and colon (100×, 100×, and 50×) of transplanted animals, as illustrated in correlation with the clinical grade of transplanted animals. Mice showing different grades of aGVHD were sacrificed throughout the experiments, i.e., at different time points. For example, recipient mice from fully allogeneic experiments were usually sacrificed between day 5 and day 15 and those from semi-allogeneic experiments between day 10 and day 30. Black lines indicate 100 µm **(B–D)** clinical grade (*x* axis) and histological grade corresponding to the sum of scores calculated for each target organ (*y* axis) are represented. Fully allogeneic combination: one experiment, *n* = 20; semi-allogeneic combination: three experiments, *n* = 23; minor-antigen mismatch combination: one experiment, *n* = 8. **(E)** Correlation between clinical and histological scores of graft-versus-host disease (GVHD) after compiling all the data in all the donor–recipient genetic combinations. *r* = Spearman’s correlation coefficient.

Three different models of mismatched BM transplantation were performed: fully allogeneic (B6 → C3H females); semi-allogeneic (B6 → B6C3F1 females); and minor-antigen mismatch (BALB/c → D2 females). Recipient mice were lethally irradiated and then received freshly isolated BM cells with CD3^+^ Tconvs at day 0 to induce aGVHD. In the fully or semi-allogeneic combinations in our hands, all mice died with characteristic clinical signs of aGVHD by day 30 and day 45, respectively (not shown). When donors and recipients differed only for minor antigens, the defining clinical signs of aGVHD mainly included hunching posture, skin lesions, and dull fur, but not weight loss or diarrhea. Mice had to be sacrificed at day 45 due to prostrated posture and consequently for ethic reasons. Clinical scores of aGVHD were calculated three times per week according to Section “Materials and Methods,” starting from day 10 after transplant. Indeed, we estimated that clinical signs occurring before day 10 were mostly induced by the conditioning regimen rather than by aGVHD. Of note, in mice transplanted with BM alone, we never detected any clinical signs of GVHD and 100% of them survived until day 60 (*n* = 25). To correlate the clinical observations with the histopathological signs of the disease, mice showing different grades of aGVHD were sacrificed (i.e., at different time points), and target organs of aGVHD (skin, liver, small intestine and colon) (22) were analyzed by a pathologist blinded to the nature of the mice being examined, to assess the intensity of aGVHD as illustrated (Figure 1A). When we looked at each target organ, in the fully allogeneic combination, we observed a clear correlation between clinical scores and histopathological scores in the liver, small intestine, and colon. Regarding the skin, the histopathological scores were constantly high, irrespective of the clinical score (Figure S1 in Supplementary Material). In the semi-allogeneic combination, a good correlation was observed in the skin, and colon, and to a lesser degree in the liver (Figure S2 in Supplementary Material). In aGVHD due to minor-antigen disparities between donors and recipients, we observed quite modest signs of aGVHD in all studied target organs (Figure S3 in Supplementary Material). Importantly, when summing up the scores of all target organs of aGVHD for each mouse, we observed a complete correlation between our new clinical grading system and the histological scores in all of the three combinations (Figures 1B–D) or in a mix of mice regardless of their genetic background (Figure 1E). Such a correlation was observed for high, moderate, or low histologic aGVHD characterization, i.e., the higher the clinical score, the higher the histological score. These data suggest that our grading system provides a complete and precise evaluation of the clinical lesions of aGVHD. This grading scale can be applied all along the duration of the experiment and the disease, in a complete and constant correlation with its histological counterpart.

### The Clinical Grading System Is Predictive of Mice Survival

**Figure 2:**
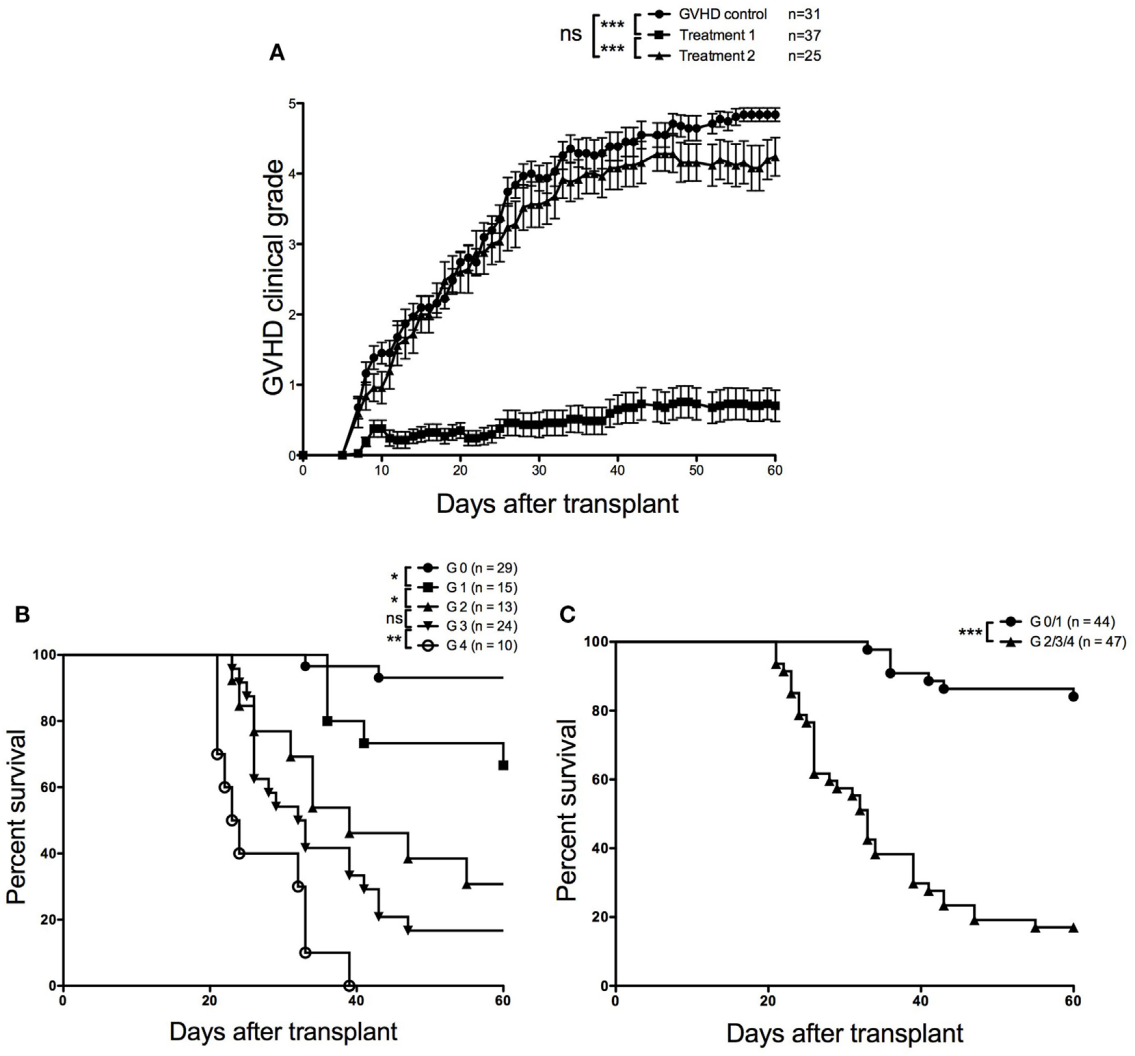
Retrospective meta-analysis from 10 independent experiments. **(A)** Evolution of clinical scores over time for each group of recipient mice receiving (i) donor bone marrow (BM) cells + conventional T cells (Tconvs) [graft-versus-host disease (GVHD) control group] or (ii) donor BM cells + Tconvs + regulatory T cells (Tregs) (treatment 1) or (iii) donor BM cells + Tconvs + Tregs + anti-TNFR2 (treatment 2), **(B)** survival curves according to clinical acute graft-versus-host disease GVHD (aGVHD) grading performed at day 20 posttransplant (*P* = 0.0248 for grade 0 versus grade 1; *P* = 0.0259 for grade 1 versus grade 2; *P* = 0.2606 for grade 2 versus grade 3; and *P* = 0.0070 for grade 3 versus grade 4). **(C)** Survival curves established for mice displaying no or mild (grade 0–1) versus moderate to severe (grade 2–4) clinical aGVHD at day 20 posttransplant (*P* < 0.0001). Survival and clinical scoring data were compiled from 10 aGVHD experiments including 91 transplanted mice.

To assess the capacity of our grading system to predict mice survival after allo-HSCT, we used our experimental model in which aGVHD was prevented by transfer of therapeutic donor Tregs (18). In a retrospective meta-analysis, we merged clinical (Figure 2A) and survival (Figures 2B,C) data from 10 independent experiments in which 93 mice received (i) donor BM cells + Tconvs (GVHD control group) or (ii) donor BM cells + Tconvs + Tregs (treatment 1) or (iii) donor BM cells + Tconvs + Tregs + anti-TNFR2 (treatment 2), an additional treatment that inhibited Treg effect, as previously reported (19). We evaluated the survival of mice according to their clinical score of aGVHD calculated at day 20 posttransplant (mice with a clinical score of 5 or dead at day 20 or before were excluded), irrespective of their assigned treatment. This time cutoff point was selected because, as shown by our previous experiments using this model, the first symptoms of aGVHD usually start to develop around day 15. Our results reveal a remarkable difference in survival between each grade (Figure 2B). Indeed, the survival percentages at the end of the experiments, i.e., at day 60 posttransplant, are 93, 67, 31, 17, and 0% for mice graded 0, 1, 2, 3, and 4 at day 20, respectively. When comparing the survival curves 2 by 2, all differences are statistically significant, except for the comparison between mice graded 2 and those graded 3 at day 20 (*P* = 0.2606). Median survival was not reached for mice graded 0 or 1, was 39 days for grade 2, 32.5 days for grade 3, and 23.5 days for grade 4. As survival dramatically worsened between mice graded 1 and those graded 2, and to simplify and dichotomize our prognostic tool, we separated mice into two groups: no/mild aGVHD (grade 0 or 1) versus moderate to severe aGVHD (grade 2 or more) at day 20 (Figure 2C). Median survival was not reached for the first group and was 33 days for mice graded 2 or higher. The corresponding survival rates at day 60 were 84 and 17%, respectively. Statistical difference between the two survival curves was highly significant (*P* < 0.0001). Importantly, these two curves also efficiently differentiate mice that received therapeutic Treg (86% of mice graded 0 or 1 at day 20) from mice that did not or that received the inhibitory treatment (anti-TNFR2). Taken together, these results prove that our clinical grading system efficiently predicts survival of mice early after transplant.

### The Clinical Grading System Is Reproducible among Experimenters

**Figure 3:**
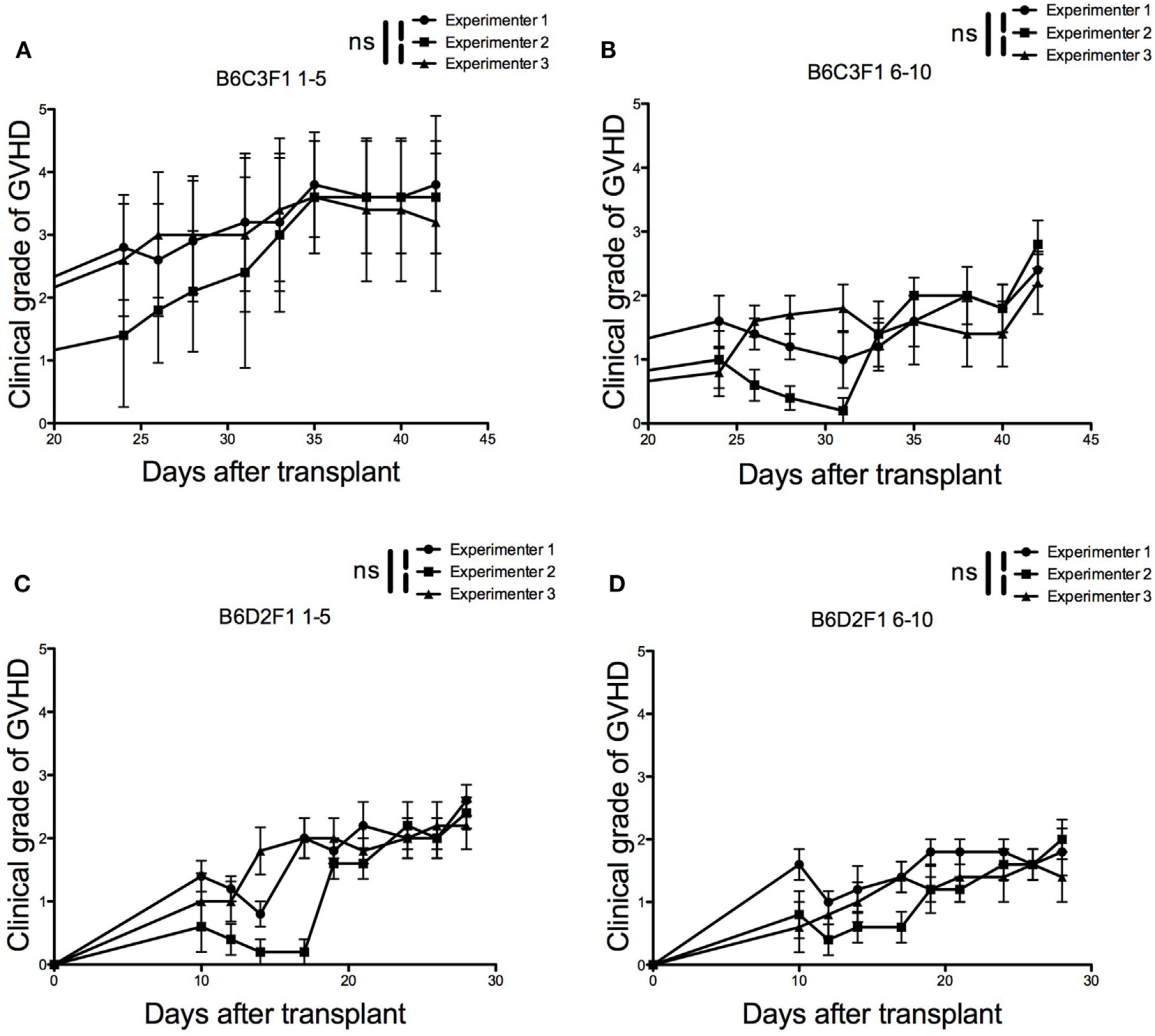
Reproducibility of the grading system among different experimenters. Three different experimenters independently scored acute graft-versus-host disease during 20 days in four independent experiments performed in two different genetic combinations (*n* = 5 for each experiment). **(A)** Recipient B6C3F1 mice received 2 × 10^6^ CD3^+^ cells + 10 × 10^6^ bone marrow (BM) cells from B6 donor mice. **(B)** Recipient B6C3F1 mice received 2 × 10^6^ CD3^+^ cells + 10 × 10^6^ BM cells from previously protected B6C3F1 mice that had undergone primary semi-allogeneic transplantation from B6 donor mice in the presence of regulatory T cells (Tregs). **(C)** Recipient B6D2F1 mice received 2 × 10^6^ CD3^+^ cells + 10 × 10^6^ BM cells from B6 donor mice. **(D)** Recipient B6D2F1 mice received 2 × 10^6^ CD3^+^ cells + 10 × 10^6^ BM cells from previously protected B6C3F1 mice that had undergone primary semi-allogeneic transplantation from B6 donor mice in the presence of Tregs.

One of the main drawbacks of the currently used grading systems is that by dividing each criterion into several severity stages according to mostly subjective parameters, the global score calculated for each mouse is highly dependent on the experimenter, and its reproducibility is at least uncertain. To test the reproducibility of our approach, we selected two models of semi-allogeneic BM transplantation (B6 → B6C3F1 females and B6 → B6D2F1 females) and performed four independent experiments (Figure S4 in Supplementary Material). Three separate experimenters, of whom two were completely blinded to the nature of the mice being examined, graded the mice three times a week using our clinical grading system. Experimenter 1 (Sina Naserian) was the first author of this manuscript whereas both experimenters 2 and 3 had limited or no experience in grading aGVHD. A fourth experimenter trained experimenters 2 and 3 to score aGVHD. Our results show very similar clinical scores over time in both genetic combinations, with a degree of concordance that even seemed to increase as aGVHD lesions got fully established, suggesting the high reproducibility of our grading system (Figure 3).

## Discussion

The clinical grading system developed by Cooke et al. in 1996 (8) can be considered as the reference of murine aGVHD assessment, having been used directly or with minor adaptations by multiple research teams worldwide during the last two decades, in a multiplicity of aGVHD models and mice combinations. However, neither the reproducibility of this grading system and its derivatives nor its correlation to histological findings and capacity to predict survival has ever been properly tested. We also believe that it could be improved in terms of ease of use, reproducibility, and completeness of the clinical features assessed.

The key point of the adaptation of Cooke’s scoring system that we propose is its simplicity (yes or no) that facilitates the reproducibility among different experimenters. To the best of our knowledge, this is the first time that the reproducibility of a murine clinical grading system has been properly tested and demonstrated. Of course, one could argue that using this scoring system three times per week could be time consuming. We used this frequency to strongly validate the relevance of our system over time, but we recommend using it once to twice a week in routine practice.

We also demonstrated that our grading system strongly correlates with histological scoring, regardless of allogeneic diversities or genetic background of the mice. This also renders the complete evaluation of aGVHD possible *via* visual parameters that neatly reflect the complexity of the disease without the need to sacrifice the mice. Interestingly, as observed in this work, the disease can reach different target organs depending on the genetic combination that is used (see Figures S1–S3 in Supplementary Material); yet our grading system appears unaffected by these disparities. Rather, as illustrated by the minor-antigen mismatch BALB/c → D2 combination, it enabled us to collect further clinical information in a combination in which weight loss and survival of recipient mice, the two mostly used criteria, were not affected by aGVHD. For this reason and to stay in a simple binary “yes or no” system for each criterion, we chose not to impact the score by the intensity of the weight loss. It should be underlined that this loss of information does not seem to affect either the correlation with the histological signs or the ability of our system to predict very quickly the outcome of mice. Indeed, when applying this scoring system at day 20 posttransplant without considering the treatment administered to recipient mice, we were able to strongly predict survival of recipient mice.

Of course, our grading system has several limitations. First of all, it could raise concerns about the relative lack of specificity of most of the criteria used to evaluate clinical aGVHD (hunching, dull hair, and weight loss). Indeed, one may argue that some of them, if not all of them, could also be a consequence of pretransplant irradiation for example. To avoid this bias, we started grading our recipient mice from day 10 posttransplant, as most of the clinical manifestations related to irradiation tend to resolve between day 5 and day 10 in our model. However, depending on the conditioning regimen used and the genetic combination between donor and recipient mice, the time frame of occurrence of clinical signs and symptoms of aGVHD may vary and overlap with the one of irradiation-related symptoms.

Moreover, as the prognostic capacity of our score calculated at day 20 was evaluated thanks to a retrospective analysis of previously performed experiments; experimenters were unblinded to the treatment received by each mouse. Hence, a judgment bias cannot be ruled out. Whereas the purpose of this work was to validate a simple and reproducible scoring system against the gold standard of histological evidence of GVHD, it will be interesting for future work to compare the different scoring systems in a prospective fashion and determine the most fitted for GVHD assessment depending on the GVHD experimental settings and the scientific goal of the work.

Finally, just like other previously published clinical scores (8–15), our grading system does not directly evaluate clinical signs of liver aGVHD, which is virtually impossible to do in mice. Hence, research teams working specifically on this form of aGVHD will still need to resort to histopathology and/or biochemical analyses (20). However, it is interesting to note that our grading system is still correlated to histological lesions in the liver.

Moreover, besides clinical and histological assessment, it should be emphasized that other approaches have been used to evaluate and validate aGVHD experimental models, such as target tissue based RT-PCR measurements of inflammatory cytokines or chemokines (23), or circulating biomarkers of aGVHD such as ST2 (24) or regenerating islet-derived protein 3 γ (RegIIIγ) (25).

In conclusion, we report a new clinical grading system for murine aGVHD, adapted from the one described by Cooke et al. (8), to overcome some of its limitations. For the first time, we show that such a scoring system, apart from being simple, is highly reproducible, correlated to the severity of histological lesions of aGVHD and predictive of survival. If adopted by other research teams, we believe this comprehensive and transversal grading system could be used as an accurate tool to compare results acquired by different teams and become a new standard for murine aGVHD evaluation.

## Ethics Statement

All experimental protocols were approved by the local ethics committee (authorization number 11/12/12-5B) and are in compliance with European Union guidelines.

## Author Contributions

SN and ML contributed equally to this work and are first coauthors. SN, ML, SM, and JC designed the experiments. SN, ML, AT, and CP performed the experiments. CLB and YB supervised and performed irradiation procedures. FC performed all histological analyses. SN, ML, and JC performed the statistical analyses and wrote the first draft of the manuscript. All the authors revised the manuscript and agreed to submit it for publication.

## Conflict of Interest Statement

The authors declare that the research was conducted in the absence of any commercial or financial relationships that could be construed as a potential conflict of interest.

## Supplementary Material

Figure S1 Correlation between clinical and histological scores of acute graft-versus-host disease GVHD (aGVHD) in fully allogeneic HSCT. Histopathological scores of aGVHD were assessed by a pathologist in a blinded fashion in skin, liver, small intestine, and colon of transplanted animals. Clinical grade (*x* axis) and histological grade (*y* axis) for each organ are represented.

Figure S2 Correlation between clinical and histological scores of acute graft-versus-host disease (aGVHD) in semi-allogeneic HSCT. Histopathological scores of aGVHD were assessed by a pathologist in a blinded fashion in skin, liver, small intestine, and colon of transplanted animals. Clinical grade (*x* axis) and histological grade (*y* axis) for each organ are represented.

Figure S3 Correlation between clinical and histological scores of acute graft-versus-host disease (aGVHD) in minor Ag disparity allogeneic hematopoietic stem cell transplantation. Histopathological scores of aGVHD were assessed by a pathologist in a blinded fashion in skin, liver, small intestine, and colon of transplanted animals. Clinical grade (*x* axis) and histological grade (*y* axis) for each organ are represented.

Figure S4 Survival curves related to the reproducibility of the grading system among different experimenters. Three different experimenters independently scored acute graft-versus-host disease during 20 days in four independent experiments performed in two different genetic combinations (*n* = 5 for each experiment). **(A)** Recipient B6C3F1 mice received 2 × 10^6^ CD3^+^ cells + 10 × 10^6^ bone marrow (BM) cells from B6 donor mice. **(B)** Recipient B6C3F1 mice received 2 × 10^6^ CD3^+^ cells + 10 × 10^6^ BM cells from previously protected B6C3F1 mice that had undergone primary semi-allogeneic transplantation from B6 donor mice in the presence of regulatory T cells (Tregs). **(C)** Recipient B6D2F1 mice received 2 × 10^6^ CD3^+^ cells + 10 × 10^6^ BM cells from B6 donor mice. **(D)** Recipient B6D2F1 mice received 2 × 10^6^ CD3^+^ cells + 10 × 10^6^ BM cells from previously protected B6C3F1 mice that had undergone primary semi-allogeneic transplantation from B6 donor mice in the presence of Tregs.
