# Supplementary figures and images for: Simple, Reproducible, and Efficient Clinical Grading System for Murine Models of Acute Graft-versus-Host Disease
Source: Front Immunol. 2018 Jan 22;9:10. doi: 10.3389/fimmu.2018.00010 (PMC5786520; doi:10.3389/fimmu.2018.00010)

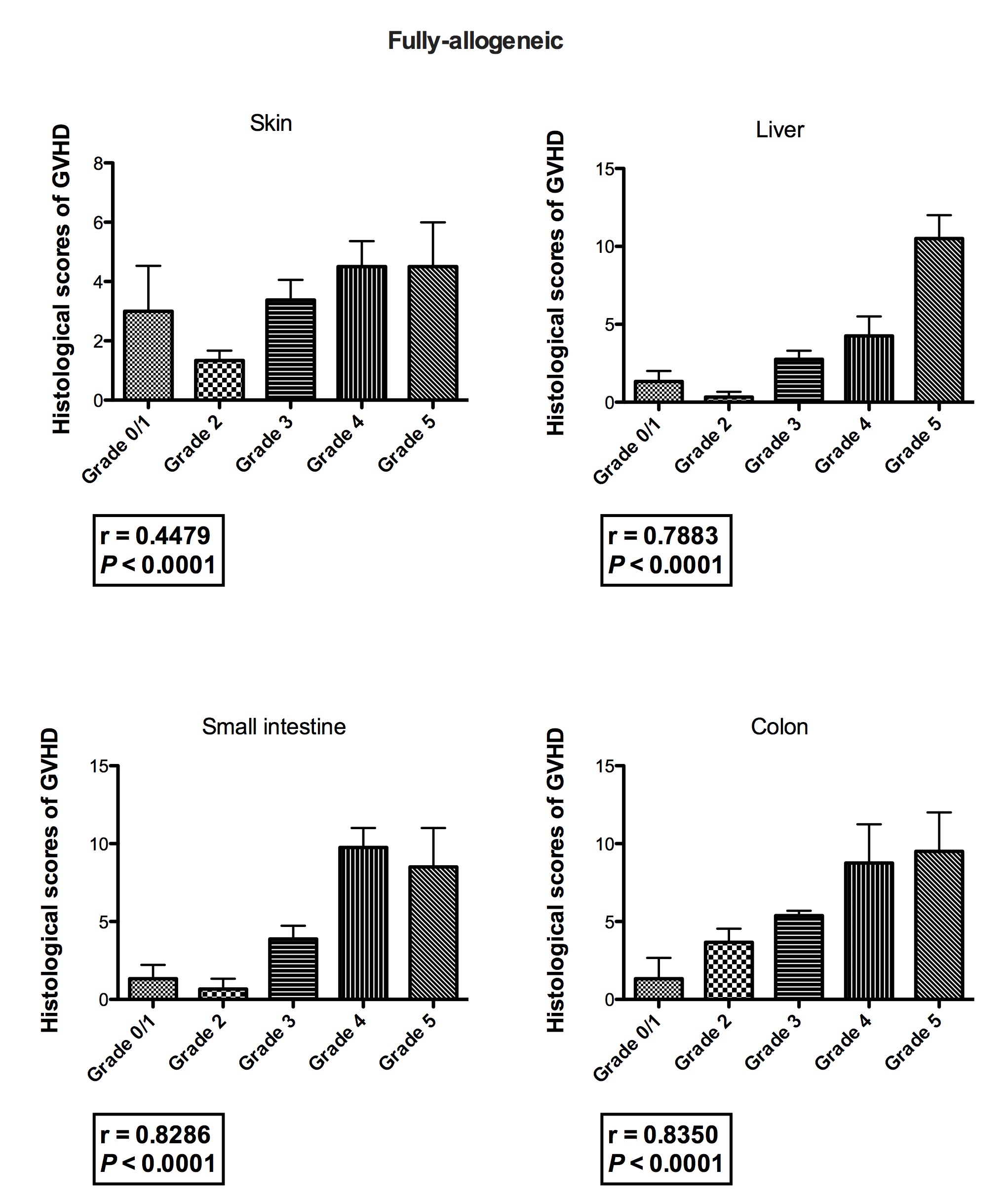

Supplement: Figure S1 — Correlation between clinical and histological scores of acute graft-versus-host disease GVHD (aGVHD) in fully allogeneic HSCT. Histopathological scores of aGVHD were assessed by a pathologist in a blinded fashion in skin, liver, small intestine, and colon of transplanted animals. Clinical grade (x axis) and histological grade (y axis) for each organ are represented. [file Image_1.jpeg]

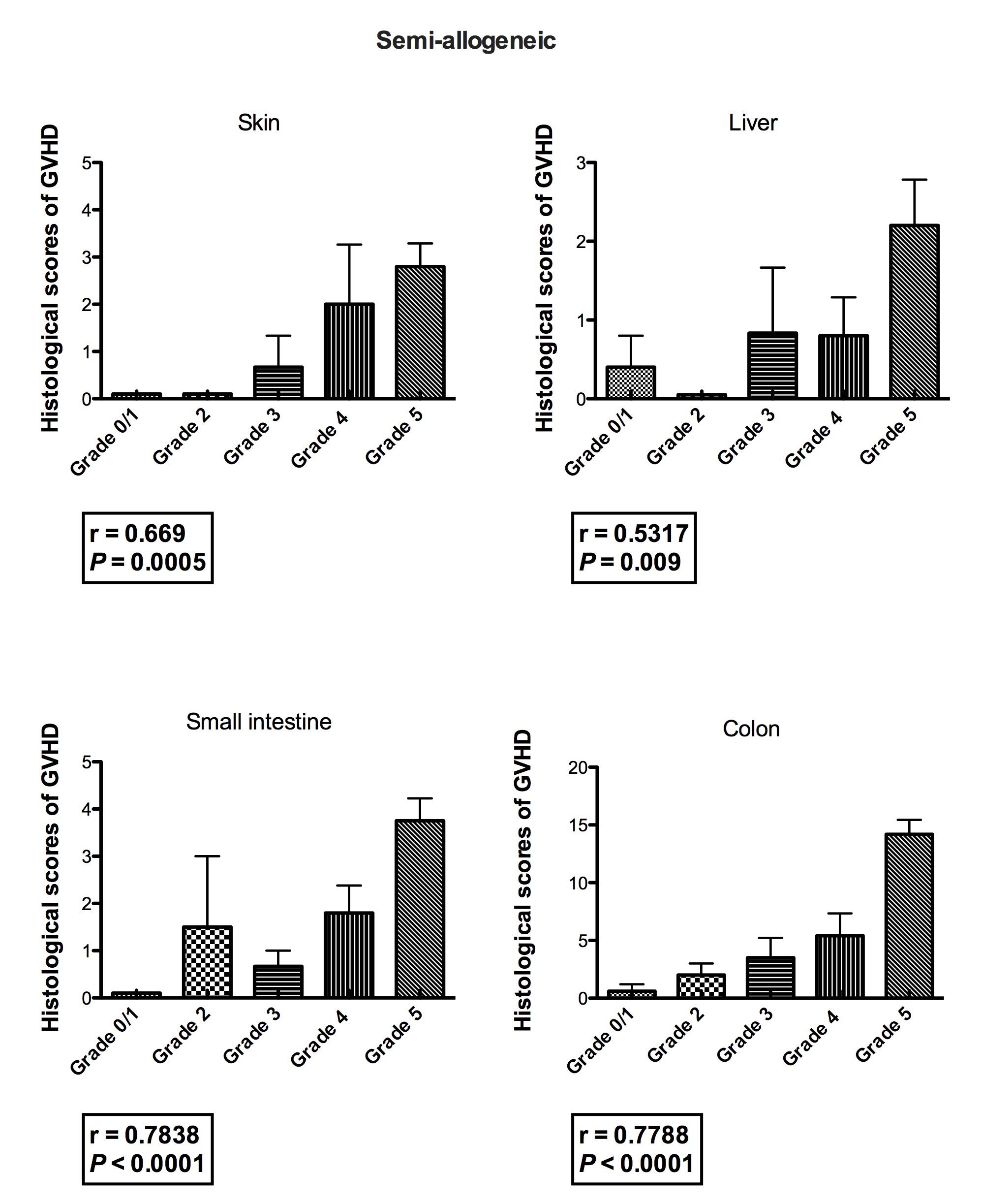

Supplement: Figure S2 — Correlation between clinical and histological scores of acute graft-versus-host disease (aGVHD) in semi-allogeneic HSCT. Histopathological scores of aGVHD were assessed by a pathologist in a blinded fashion in skin, liver, small intestine, and colon of transplanted animals. Clinical grade (x axis) and histological grade (y axis) for each organ are represented. [file Image_2.jpeg]

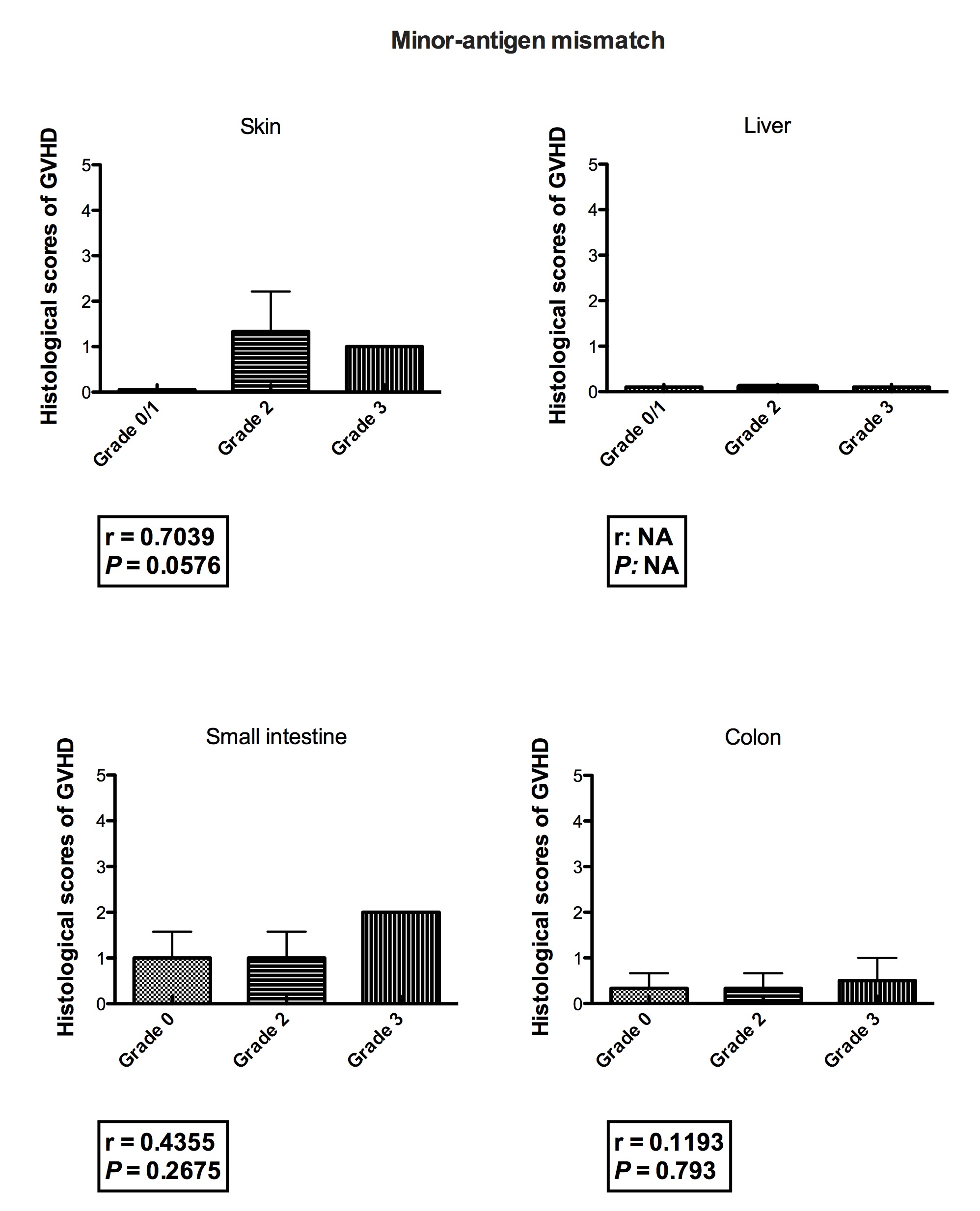

Supplement: Figure S3 — Correlation between clinical and histological scores of acute graft-versus-host disease (aGVHD) in minor Ag disparity allogeneic hematopoietic stem cell transplantation. Histopathological scores of aGVHD were assessed by a pathologist in a blinded fashion in skin, liver, small intestine, and colon of transplanted animals. Clinical grade (x axis) and histological grade (y axis) for each organ are represented. [file Image_3.jpeg]

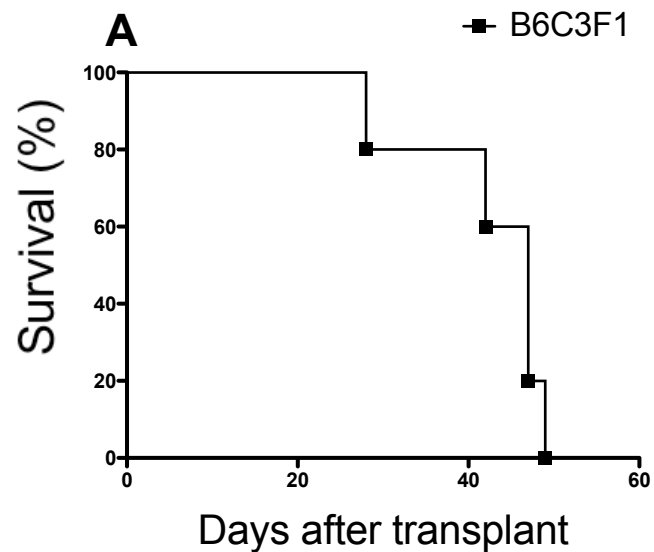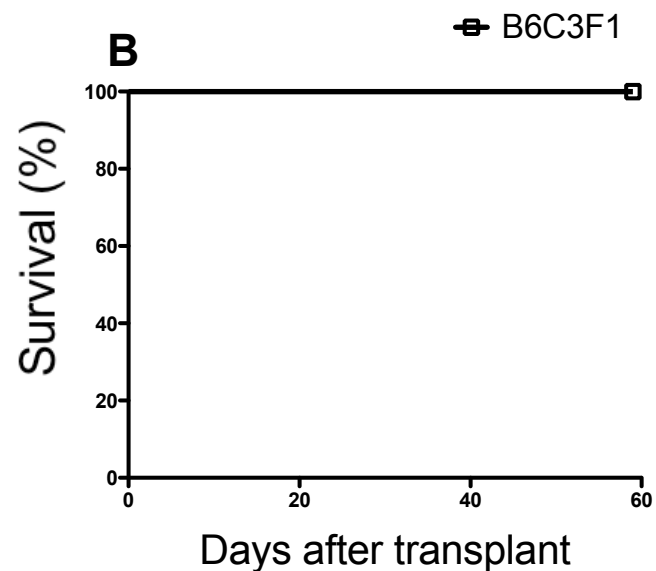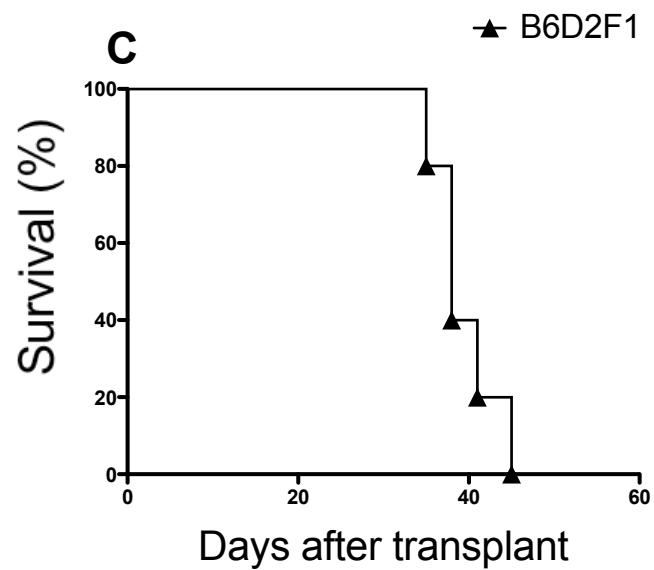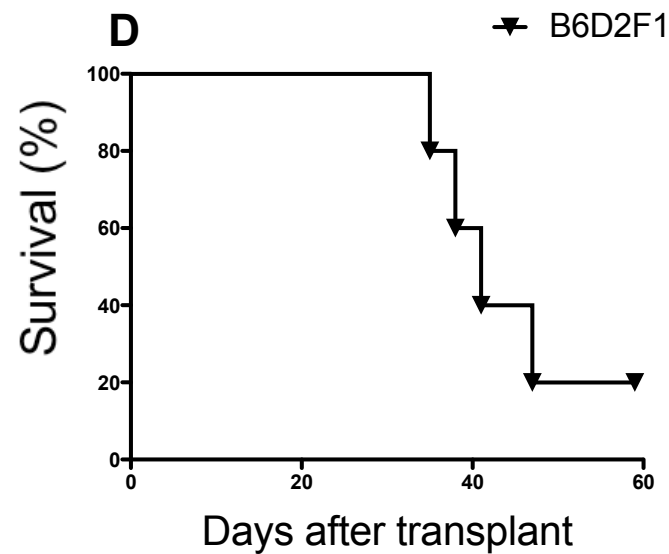

Supplement: Figure S4 — Survival curves related to the reproducibility of the grading system among different experimenters. Three different experimenters independently scored acute graft-versus-host disease during 20 days in four independent experiments performed in two different genetic combinations (n = 5 for each experiment). (A) Recipient B6C3F1 mice received 2 × 10^6^ CD3+ cells + 10 × 106 bone marrow (BM) cells from B6 donor mice. (B) Recipient B6C3F1 mice received 2 × 10^6^ CD3+ cells + 10 × 106 BM cells from previously protected B6C3F1 mice that had undergone primary semi-allogeneic transplantation from B6 donor mice in the presence of regulatory T cells (Tregs). (C) Recipient B6D2F1 mice received 2 × 10^6^ CD3+ cells + 10 × 106 BM cells from B6 donor mice. (D) Recipient B6D2F1 mice received 2 × 10^6^ CD3+ cells + 10 × 106 BM cells from previously protected B6C3F1 mice that had undergone primary semi-allogeneic transplantation from B6 donor mice in the presence of Tregs. [file Image_4.PDF]
